# Positive Bubble Study But No Evidence of Interatrial Defect in a Patient with Recurrent Cryptogenic Stroke

**DOI:** 10.4274/TJAR.2022.221106

**Published:** 2023-06-16

**Authors:** Nika Samadzadeh Tabrizi, Perry A. Stout, Joseph Cahill, Imran Ramzan Sunesara, Patrick Chan, Chanderdeep Singh, Thomas Fabian, Alexander D. Shapeton, Sridhar Reddy Musuku

**Affiliations:** 1 Albany Medical College, New York, United States; 2 Department of Cardiothoracic Surgery, Albany Medical Center, New York, United States; 3 Houston Methodist Debakey Heart & Vascular Center, Houston Methodist Hospital, Houston, United States; 4 Department of Anaesthesia, Critical Care and Pain Medicine, Tufts University Faculty of Medicine, Boston, United States; 5 Department of Anaesthesiology and Perioperative Medicine, Albany Medical Center, New York, United States

**Keywords:** Arteriovenous malformation, echocardiography, ischemic stroke, patent foramen ovale, saline contrast study

## Abstract

Pulmonary arteriovenous malformations (PAVMs) can be asymptomatic or result in a range of complications such as brain abscesses or cryptogenic emboli, which can contribute to morbidity and mortality if not diagnosed and treated in a timely manner. To date, there have been several reports of delayed diagnosis of PAVMs, which have been largely attributed to the misconception that PAVMs are too rare to be of clinical significance. Furthermore, because intracardiac shunting secondary to a patent foramen ovale (PFO) or atrial septal defect (ASD) also results in a positive saline contrast study with echocardiography, PAVM can be easily misdiagnosed as an intracardiac right-toleft shunt. However, there are unique echocardiographic features that differentiate between intracardiac shunting due to a PFO or ASD and extracardiac shunting such as in PAVM. This case details the course of a patient with recurrent cryptogenic strokes that was initially misattributed to a PFO and was only correctly diagnosed with multiple PAVMs after two failed attempts at PFO closure. This case serves as a reminder of an alternative etiology of right-to-left shunt and its presentation on imaging, which echocardiographers must be familiar with.

**Main Points**

• Pulmonary arteriovenous malformations (PAVMs) are commonly misdiagnosed, resulting in significant delays in treatment.

• In the clinical setting, PAVMs are not a rare phenomenon, even in patients without any known associated risk factors.

• Expertise in echocardiography in patients with a positive saline contrast study can improve timely diagnosis in this patient population

## Introduction

Despite what was previously thought, pulmonary arteriovenous malformations (PAVMs) are relatively common with an estimated prevalence of 1 in 2,600.^1,2^ Depending on the degree of shunting, patients can be asymptomatic or suffer from multisystem complications.^1,3^ PAVMs increase the risk of cryptogenic stroke by as much as 25% and have a 25-50% risk of mortality if left untreated.^3,4^ Alarmingly, the median delay from cerebral event to diagnosis of PAVM and from diagnosis of PAVM to referral for treatment is 2 and 7.5 years, respectively.^1,2^ Despite their relative prevalence, PAVMs remain under-recognized in physician education, resulting in misdiagnosis, delayed treatment, and increased morbidity and mortality.^2,3,4^

## Case Presentation

After obtaining informed consent, this report presents a 50-year-old man with Gilbert’s syndrome, alpha-1-antitrypsin deficiency, and chronic obstructive lung disease who initially presented to an outside institution with a left cerebellar infarct. At that time, a saline contrast study was positive for a right to left shunting (RLS), raising suspicion for a patent foramen ovale (PFO) (Video 1). Percutaneous closure was attempted, but interventionalists were unable to traverse the PFO and as a result, the patient was managed with clopidogrel. During the subsequent year, the patient suffered from recurrent strokes and was referred for surgical closure. At our institution, intraoperative transesophageal echocardiography (TEE) did not reveal an interatrial defect but agitated saline injection during Valsalva maneuver was positive for contrast in the left atrium (LA) within 2 cardiac cycles. The coronary sinus (CS) appeared normal, and the surgical team remained suspicious for a PFO. Subsequently, the patient was placed on cardiopulmonary bypass and a right atriotomy was performed. Surgical visualization of the right atrium revealed an intact fossa ovalis with an opening that was much smaller than anticipated without frank interatrial communication. Examination of the interatrial septum, CS, and vena cava was also unremarkable, prompting consultation with a pediatric cardiothoracic surgeon who confirmed these findings. Post-bypass saline contrast study remained positive with a somewhat delayed appearance of the contrast in the LA from the right pulmonary vein (Video 2), raising suspicion for a PAVM.

**Figure 1:**
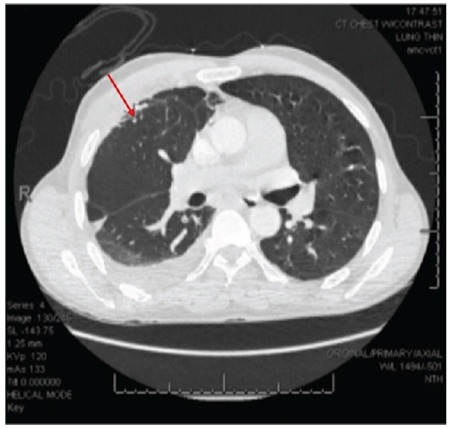
Axial computed tomography with intravenous contrast showing a pulmonary arteriovenous malformation in the right upper lobe measuring 3 mm.

**Figure 2:**
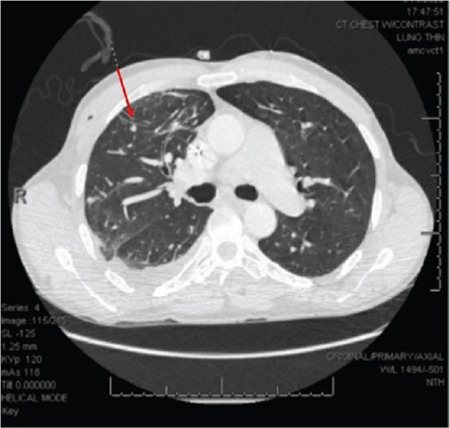
Axial computed tomography with intravenous contrast showing a pulmonary arteriovenous malformation in the right upper lobe measuring 5 mm.

**Figure 3:**
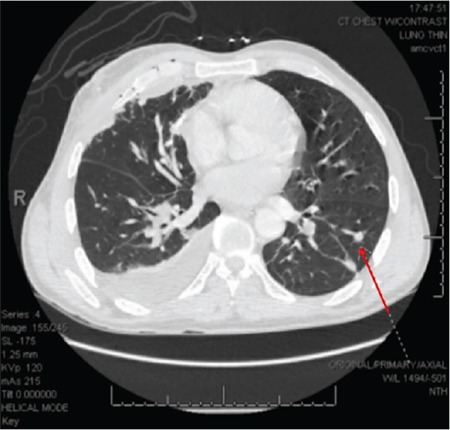
Axial computed tomography with intravenous contrast showing a pulmonary arteriovenous malformation in the left lower lobe measuring 7 mm.

**Figure 4:**
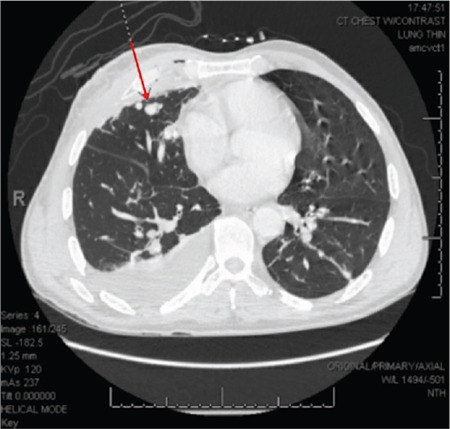
Axial computed tomography with intravenous contrast showing a pulmonary arteriovenous malformation in the right middle lobe PAVM measuring 9 mm. PAVM, pulmonary arteriovenous malformation.

Upon examination of the right lung, two prominent PAVMs were identified (Video 3), prompting consultation with a thoracic surgeon who performed wedge resection. Subsequent TEE revealed persistence of a positive saline contrast study, with flow primarily originating from the left pulmonary veins, suggesting additional PAVMs in the contralateral left lung (Video 4). At this time, the surgical team closed the incisions and planned postoperative imaging and potential coiling. Postoperative chest computed tomography (CT) with contrast revealed PAVMs in the left lower, right upper, and right middle lobes (Figures 1, 2, 3, 4). The patient was subsequently referred to interventional radiology.

## Discussion

While intracardiac shunting is most commonly the source of paradoxical emboli, it is also necessary to recognize PAVM as an alternative cause. Misdiagnosis dramatically changes the approach to treatment, resulting in delayed care.^5,6^ Left untreated, mortality associated with PAVM complications may reach 50%, especially in patients with multiple PAVMs and large feeding arteries.^4^ Those with feeding arteries greater than 2.0 to 3.0 mm in diameter are usually treated with percutaneous transcatheter embolization, while larger PAVMs with multiple feeding arteries are treated with thoracoscopic resection.^1,3,5,7^ Despite this, long-term follow-up is imperative in this patient population due to possible collateralization and recurrence.^1,8,9^

While PAVMs are most commonly associated with an autosomal dominant condition called hereditary hemorrhagic telangiectasia (HHT), they have also been reported in association with hepatopulmonary syndrome, schistosomiasis, mitral stenosis, previous thoracic surgery, metastatic thyroid carcinoma, and congenital heart disease, with at least 15% of cases attributed to idiopathic causes.^5,9,10^ However, PAVMs in the context of non-HHT have not been extensively studied.^2,5^ The largest retrospective study of non-HHT PAVMs (n = 77) reported that 61% did not have any known associated risk factors.^9^ Despite a history of alpha-1-anti-trypsin deficiency, our patient’s hepatic function was within normal limits and he lacked any other risk factors, contributing to his delayed diagnosis.

Saline contrast study with TEE is the gold standard screening test for the evaluation of intracardiac and extracardiac RLS.^5^ In RLS due to PFO, it takes 1-2 cycles for microbubbles to appear in the LA in contrast to 3-8 cycles in the case of PAVM.^3,5,11^ Visualization of microbubbles in the pulmonary vein (Video 2) may be confirmatory for PAVM. In our patient, the bubbles appeared in the LA within 2-3 cardiac cycles (grade 1 shunt) and became more diffuse and prominent as the cardiac cycle progressed through the 7-9^th^ cardiac cycle (grade 3 shunt) (Video 1).^12^ Gradual increase in the number of microbubbles in the LA should not be seen in cases of [atrial septal defects (ASDs)/PFOs]. Our patient had a negative Valsalva and Color flow Doppler across the interatrial septum (Video 1), effectively ruling out a PFO.^4^ Further, contrast-enhanced chest CT has utility in excluding PAVMs and has a high sensitivity for those with a grade 2 or 3 echocardiographic shunt.^3,5,13^ Therefore, in cases of indeterminate shunting and evidence of high-grade shunt by TEE, further evaluation by CT scan is warranted.

The timing of bubble appearance is also dependent on cardiac output, shunt size, and concomitant intracardiac defect. The bubble appearance can be delayed in cases of a PFO with an aneurysmal atrial septum or hastened in extensive PAVMs, especially when they are located proximally to the heart.^14^ In our patient, microbubbles appeared in the LA sooner than 3 cardiac cycles, likely due to multiple PAVMs with large feeding arteries. Recently, researchers used acoustic intensity mapping to quantify the saline contrast patterns that differentiate PFOs from PAVMs.^11^ Interestingly, they found that the appearance of contrast in PAVMs has a uniquely longer wash-in/wash-out phase, resulting in a greater contrast intensity in the left-sided heart chambers during the wash-out phase. This contrasts with PFOs/ASDs in which the contrast intensity is always higher in the right-sided heart chambers.

## Conclusion

When encountering a patient with a positive saline contrast study, one must consider PAVMs as a potential source given that most PAVMs in the context of non-HHT are idiopathic and cannon be excluded in a patient with cryptogenic emboli.^5,9^ In doing so, echocardiographers must familiarize themselves with echocardiography features that distinguish between common and rare causes of RLS.

**Video 1.** Transesophageal echocardiographic saline contrast study at the time of the patient’s initial percutaneous patent foramen ovale closure attempt at an outside hospital. Mid esophageal (ME) bicaval view with and without color flow doppler demonstrating lack of interatrial communication and color flow across the interatrial septum at a color scale of 46.2. ME bicaval view of saline contrast study demonstrating appearance of microbubbles in the left atrium in 2.5 cardiac cycles. Freeze frames at 2.5 and 7^th^ cardiac cycles demonstrate that the microbubbles become progressively more prominent and diffuse later on in the cardiac cycle.

https://doi.org/10.4274/TJAR.2022.221106.video1

**Video 2.** Intraoperative transesophageal echocardiographic saline contrast studies at our institution. ME bicaval view demonstrates a delayed appearance of microbubbles in the left atrium from the right pulmonary vein. Mid esophageal coronary sinus view does not reveal any patent foramen ovale or atrial septal defect.

https://doi.org/10.4274/TJAR.2022.221106.video2

**Video 3.** Surgical view of the pulmonary arteriovenous malformation (PAVM) located in the right middle lung which was resected. The air at the top of the PAVM can be visualized due to its distinctly light-colored appearance.

https://doi.org/10.4274/TJAR.2022.221106.video3

**Video 4.** Saline contrast study performed after surgical resection of two pulmonary arteriovenous malformations. The study demonstrates a positive contrast study with reduced number of microbubbles in the left atrium, raising concern for additional sources of right-to-left shunt.

https://doi.org/10.4274/TJAR.2022.221106.video4
